# Supplementary material for: Global increase in rain rate of tropical cyclones prior to landfall
Source: Nat Commun. 2026 Jan 7;17:114. doi: 10.1038/s41467-025-68070-z (PMC12779963; doi:10.1038/s41467-025-68070-z)
Supplement: Supplementary file 1 — Supplementary Information [file 41467_2025_68070_MOESM1_ESM.pdf]

Supplementary Information

Global Increase in Rain Rate of Tropical Cyclones Prior to Landfall

Quanjia Zhong[1], Jianping Gan[1,2*], Shifei Tu[3], Ralf Toumi[4], Johnny C. L. Chan[5,6,7*]

1. *Center for Ocean Research in Hong Kong and Macau (CORE) and Department of Ocean Science, Hong Kong University of Science and Technology; Hong Kong, China*
2. *Department of Mathematics, Hong Kong University of Science and Technology; Hong Kong, China*
3. *South China Sea Institute of Marine Meteorology/Western Guangdong Key Laboratory of Marine Meteorological Disaster Theory and Application, College of Ocean and Meteorology, Guangdong Ocean University, Zhanjiang, China*
4. *Department of Physics, Imperial College London, London, UK*
5. *Asia-Pacific Typhoon Collaborative Research Center, Shanghai, China*
6. *Shanghai Typhoon Institute, China Meteorological Administration, Shanghai, China*
7. *School of Energy and Environment, City University of Hong Kong, Hong Kong, China*

**Corresponding authors*:magan@ust.hk &Johnny.Chan@cityu.edu.hk

This PDF contains Supplementary Tables 1-2 and Supplementary Figures 1-8.

Supplementary Table 1 | Statistics of tropical cyclones (TCs) rain rate with time for various hemispheres, areas, ocean basins, latitudinal belts, intensity categories, and sensitivity experiments.

	Number of TCs	Mean rain rate (mm h^{-1})	Trend (mm h^{-1} per day)	P-value	Increment (%)
**Global**	1468	1.93	0.14±0.01	< 0.001	20.42
**N. Hemisphere**	1111	1.94	0.14±0.02	< 0.001	20.02
**S. Hemisphere**	357	1.90	0.15±0.01	< 0.001	22.66
**Entire TC**	1468	1.93	0.14±0.01	< 0.001	20.42
**Inner core**	1468	3.07	0.28±0.03	< 0.001	25.88
**Outer region**	1468	1.15	0.05±0.00	< 0.001	10.81
**WNP**	600	2.14	0.18±0.02	< 0.001	23.90
**ENP**	78	1.79	0.10±0.02	< 0.001	14.56
**NA**	210	1.47	0.11±0.01	< 0.001	21.21
**SP**	150	2.05	0.11±0.01	< 0.001	14.95
**NI**	94	1.92	0.14±0.01	< 0.001	19.28
**SI**	190	1.79	0.18±0.01	< 0.001	27.99
**5° – 10°**	55	1.47	0.09±0.02	< 0.001	17.61
**10° – 15°**	267	1.73	0.19±0.01	< 0.001	31.82
**15° – 20°**	386	1.89	0.28±0.02	< 0.001	44.52
**20° – 25°**	279	2.14	0.18±0.02	< 0.001	23.67
**25° – 30°**	95	1.76	0.04±0.02	< 0.001	5.58
**30° – 35°**	117	2.28	0.11±0.03	< 0.001	12.72
**TS**	455	1.56	0.19±0.01	< 0.001	35.46
**Cat1**	199	1.98	0.25±0.02	< 0.001	36.87
**Cat2**	115	2.25	0.11±0.02	< 0.001	12.89
**Cat3**	88	2.25	0.19±0.03	< 0.001	24.15
**Cat4**	142	2.67	0.18±0.02	< 0.001	18.60
**Cat5**	61	2.95	0.40±0.03	< 0.001	40.94
**TRMM**	650	2.76	0.22±0.02	< 0.001	22.52
**ERA5**	650	2.42	0.19±0.01	< 0.001	21.14
**MSWEP**	650	1.91	0.15±0.02	< 0.001	22.35
**Experiment 1 (EXP1, Land-Rad)**	1	3.08	0.65±0.12	< 0.001	86.81
**Experiment 2 (EXP2, noLand-Rad)**	1	3.08	0.31±0.14	0.051	30.16
**Experiment 3 (EXP3, Land-noRad)**	1	3.05	0.00±0.07	0.240	0.75

The "*p*-value" represents the significance of these linear trends, and "Increment" represents the differences in average rain rate between the first and last points of the linear trend (i.e., see Methods). The trend and increment are calculated globally, in the two hemispheres [(NH: North Hemisphere, SH: South Hemisphere)], in the three sensitively experiments [Experiment 1(EXP1, Land-Rad): with land & with radiation, Experiment 2 (EXP2, noLand-Rad): without land & with radiation, and Experiment 3(EXP3, Land-noRad): with land & without radiation], in individual basins (i.e., WNP: western North Pacific, ENP: eastern North Pacific, NA: North Atlantic, SI: South Indian Ocean, SP: South Pacific, NI: North Indian Ocean), with different TC intensities (i.e., TS: tropical storm, Cat1: Category 1, Cat2: Category 2, Cat3: Category 3, Cat4: Category 4, and Cat5: Category 5) and in different latitudinal belts (5°-10°, 10°-15°, 15°-20°, 20°-25°, 25°-30°, and 30°-35°). The 5°-10° represents the landfalling latitudinal belts of 5-10°S and 5°-10°N, and similarly for 10°-15°, 15°-20°, 20°-25°, 25°-30°, and 30°-35°. We calculated mean rain rate for each grouping of TCs from 60 hours before landfall to landfall. All the data are from the Multi-Source Weighted-Ensemble Precipitation (MSWEP) dataset except for the two rows labeled TRMM and ERA5, which we used to validate the MSWEP dataset.

Supplementary Table 2 | Experiment design

Experiment numbers	The use of land	The use of radiation schemes
**Experiment 1 (EXP1, Land-Rad)**	land use type of Dryland Cropland and Pasture	RRTMG shortwave and longwave radiation schemes
**Experiment 2 (EXP2, noLand-Rad)**	without the presence of land	RRTMG shortwave and longwave radiation schemes
**Experiment 3 (EXP3, Land-noRad)**	land use type of Dryland Cropland and Pasture	without any radiative effect

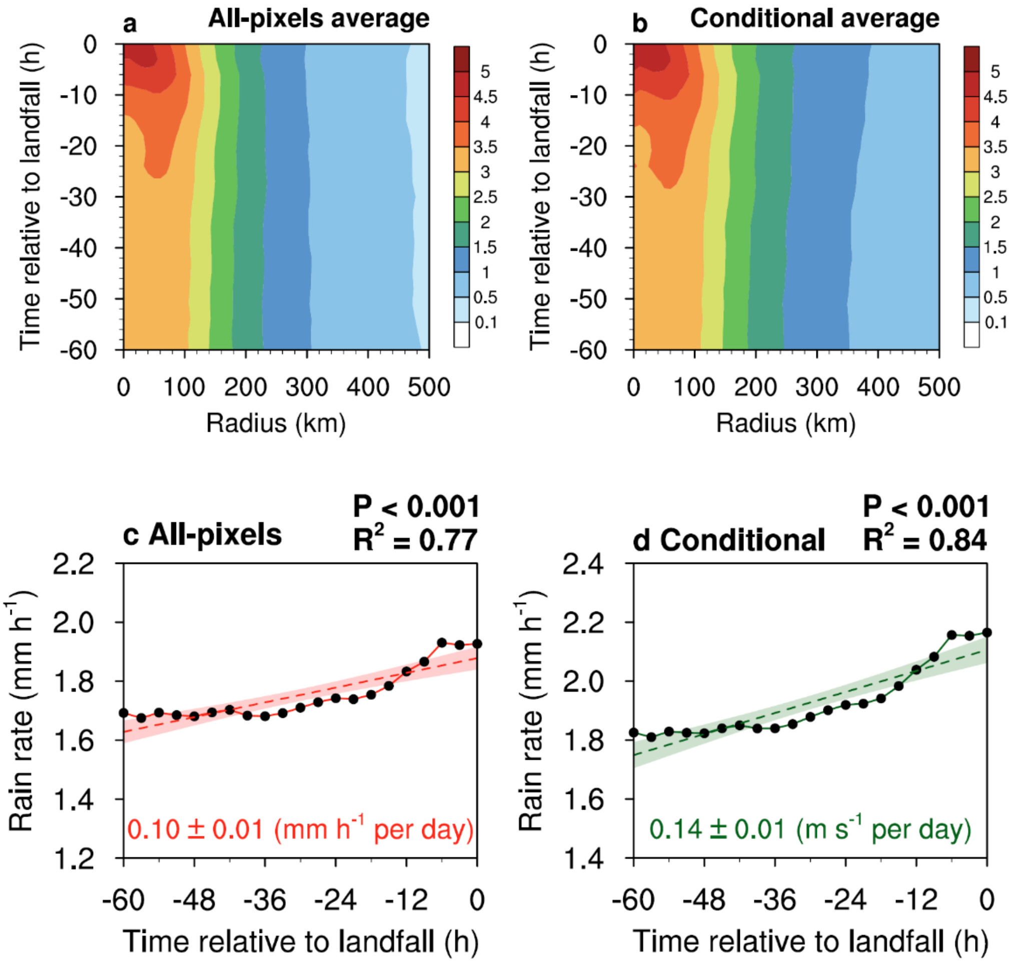

Supplementary Fig. 1 | Comparisons of the averaged rain rate and the conditional rain rate with a value larger than 0.1 mm h^{-1} of the landfalling tropical cyclones (TCs). (a), **(b)** Radius–time Hovmöller diagram and **(c)**, **(d)** Time series. We calculate the average rain rate from the data from all pixels, but the conditional rain rate only considered data points with rain rates larger than 0.1 mm h^{-1}. Statistical significance of the linear trends was assessed using a Student's t-test. Light shading in panels **(c)** and **(d)** represents the two-sided 95% confidence boundary of the linear trend. The dashed lines indicate the linear regression of the mean rain rate with time before landfall. The linear regression coefficients with error estimates (unit: mm h^{-1} per day) are at the bottom. The R^2 and p values are in the top right corner. Time is presented in hours relative to landfall time (00 h), with negative meaning hours before TC landfall.

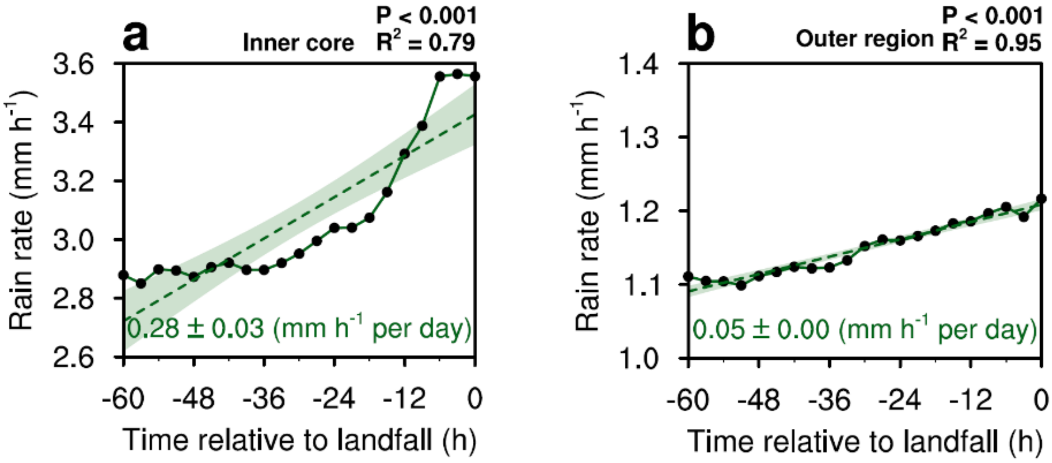

Supplementary Fig. 2 | Changes in the rain rate of landfalling tropical cyclones (TCs). The global mean rain rate of all TCs from 60 hours before landfall to the time at landfall at 3-hourly intervals (solid line with black dots) and the linear trends (dashed line) from 1980 to 2020. (**a**) Inner core (0 to 200 km) and **(b)** Outer region (200 to 500 km). Statistical significance of the linear trends was assessed using a Student's t-test. Light shading in all panels represents the two-sided 95% confidence interval of the linear trend. The dashed lines indicate the linear regression of the mean rain rate with the time before landfall. The linear regression coefficients with error estimate (unit: mm h^{-1} per day) are at the bottom. The R^2 and p values are in the top right corner. The x-axes time is in hours relative to landfall time (00 h), with negative meaning hours before landfall.

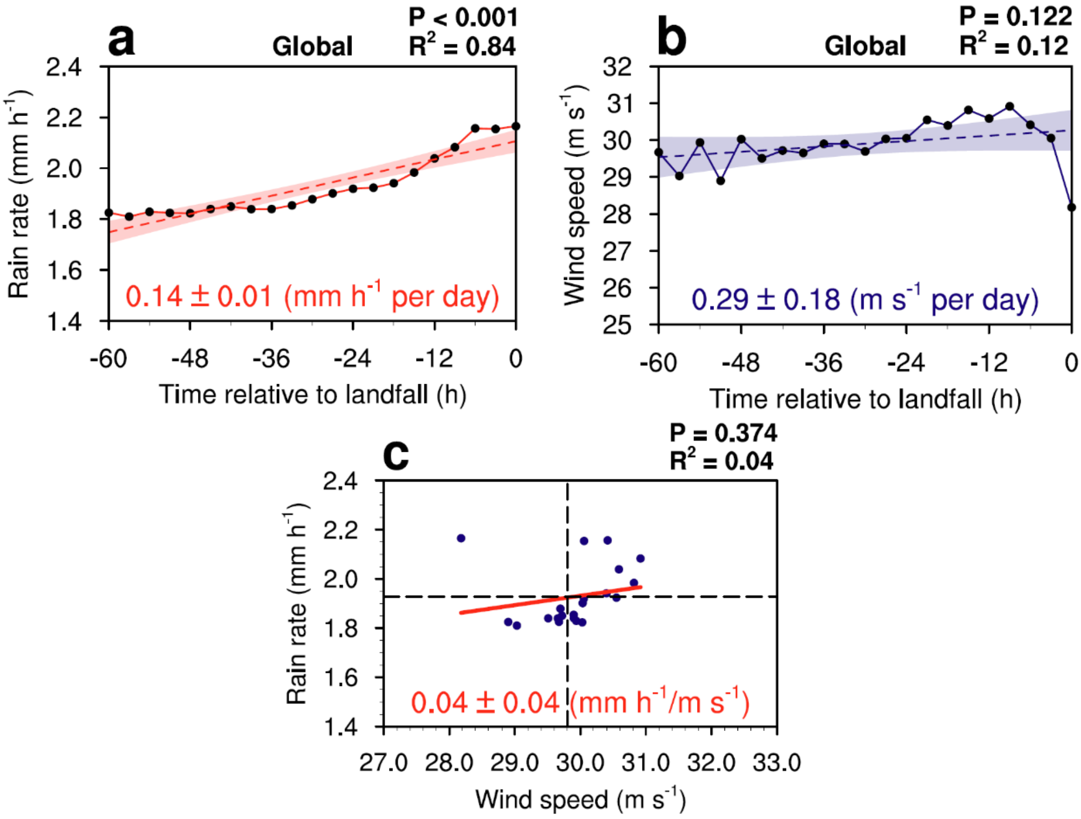

Supplementary Fig. 3 | Changes in the mean rain rate and mean intensity of landfalling tropical cyclones (TCs) globally, and their relationship. In panels (**a**) and (**b**), the mean rain rates and intensities of all TCs from 60 hours before landfall to the time of landfall at 3-hour intervals are shown by the solid line with black dots, and the linear trends are shown by the dashed line. Statistical significance of the linear trends was assessed using a Student's t-test. Light shading in all panels represents the two-sided 95% confidence interval of the linear trend. The x-axis represents time in hours relative to landfall (00 h), where negative values indicate hours before landfall. In panel (**c**), the red line indicates the linear regression between the mean rain rate and the mean intensity before landfall. The linear regression coefficients with their error estimates (unit: mm h^{-1} per day) are shown at the bottom. The R^2 and p values are in the top right corner.

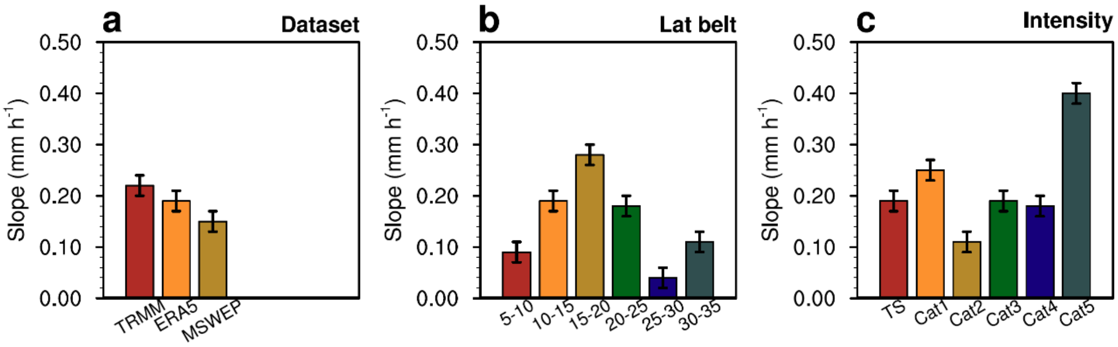

Supplementary Fig. 4 | Slopes of the mean rain rate for landfalling tropical cyclones (TCs) 60 hours before landfall and their error estimates. The mean rain rate of TCs is at 3-hourly intervals. Slopes of rain rate obtained from (**a**) datasets (MSWEP, TRMM, ERA5), (**b**) latitudinal belts (5°-10°, 10°-15°, 15°-20°, 20°-25°, 25°-30°, and 30°-35°); and **(c)** intensity categories (TS: tropical storm, Cat1: Category 1, Cat2: Category 2, Cat3: Category 3, Cat4: Category 4, and Cat5: Category 5). 5°-10° represents the landfalling latitudinal belts of 5°-10°S and 5°-10°N, and similarly for 10°-15°, 15°-20°, 20°-25°, 25°-30°, and 30°-35°.

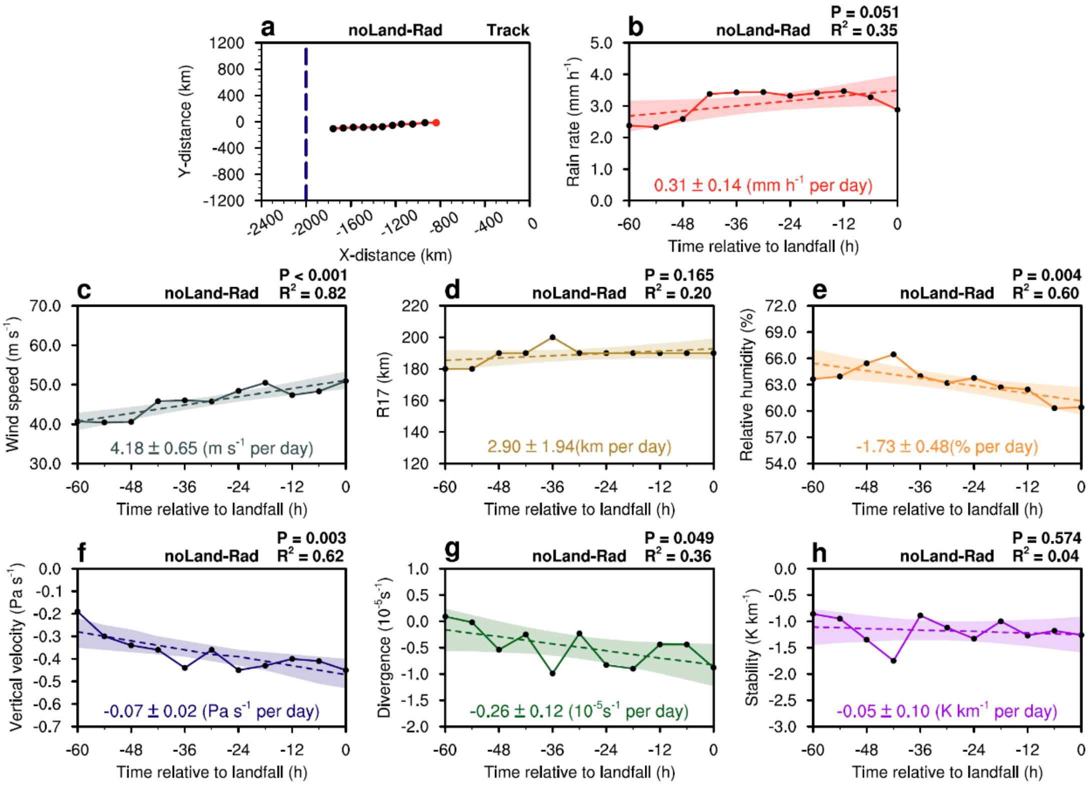

Supplementary Fig. 5 | Track and changes in rain rate, intensity, size and environmental parameters of the simulated Tropical cyclone (TC) in the Experiment 2 (EXP2, noLand-Rad). (**a**) 60-hr track, (**b**) rain rate (mm h^{-1}), (**c**) maximum wind speed (m s^{-1}), (**d**) radius of 17 m s^{-1} wind(R17, km), (**e**) relative humidity (%), (**f**) vertical velocity (Pa s^{-1}); (**g**) divergence (10^{-5} s^{-1}), and (**h**) stability (K km^{-1}) of the simulated TC at 6-hourly intervals (solid lines with black dots). Different colors of lines and shading represent different environmental parameters across the entire TC (0 to 500 km radius). In panel (**a**), the starting point is the TC location 60 hours before landfall (red dot) and the blue dashed line marks the "imaginary coastline", with the sea located to the west and to the east. Light shading in panels (**b**) to (**h**) represents the 95% confidence interval of the linear trend. Statistical significance of the linear trends was assessed using a Student's t-test. Dashed lines indicate the linear regression of the rain rate, intensity, size and environmental parameters during the time before landfall. Linear regression coefficients with corresponding standard errors are shown at the bottom, and R^2 and p values are provided in the top right corner. Time is presented in hours relative to landfall (00 h), with negative values indicating hours before TC landfall.

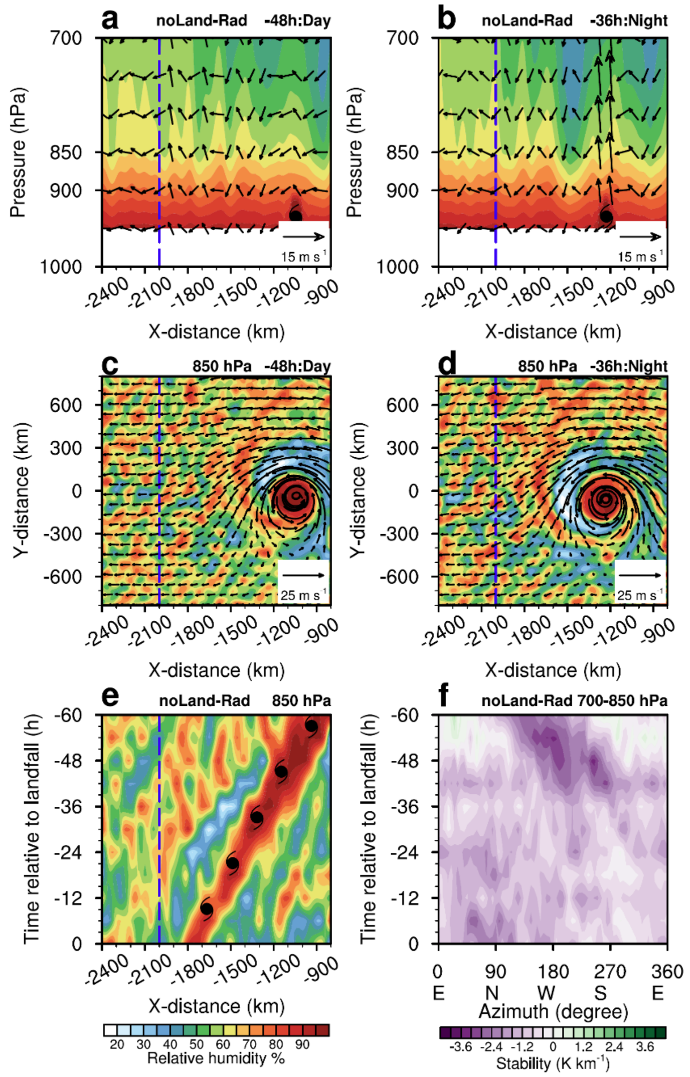

Supplementary Fig. 6 | Spatial and temporal variations of wind vectors (m s^{-1}), relative humidity (shading, %), and stability (K km^{-1}) in the Experiment 2 (EXP2, noLand-Rad). (**a**), (**b**) Radius–pressure Hovmöller diagrams of wind and relative humidity at y = 0 km for the TC during the 48 hours of daytime and 36 hours of nighttime before landfall. (**c**), (**d**) horizontal distributions of wind and relative humidity fields at 850 hPa during daytime and nighttime. (**e**), (**f**) azimuthal–time Hovmöller diagrams of respective relative humidity at 850 hPa and stability (the difference in equivalent potential temperature between 850 hPa and 700 hPa). In panels (**a**) to (**d**), the time in the top left corner is in hours relative to landfall (00 h), with negative values indicating hours before landfall. Day and night refer to horizontal and vertical distributions of wind vectors and relative humidity (shading) during the day and night, respectively. In panels (**e**) and (**f**), we averaged the azimuthal–time Hovmöller diagram over the tropical cyclone radius from 0 to 500 km. In panels (**a**) to (**e**), the blue dashed line marks the "imaginary coastline", with the sea located to the west and to the east. The radius values on the x- and y-axes are referenced to the model domain center, where negative values represent positions west and south of the center and positive values represent positions east and north of the center. In panels (**a**), (**b**), and (**e**), the typhoon symbol corresponds to the x-axis values of the TC center location. In panel (**f**), the letters E, N, W, and S represent East, North, West, and South in Earth's coordinate system.

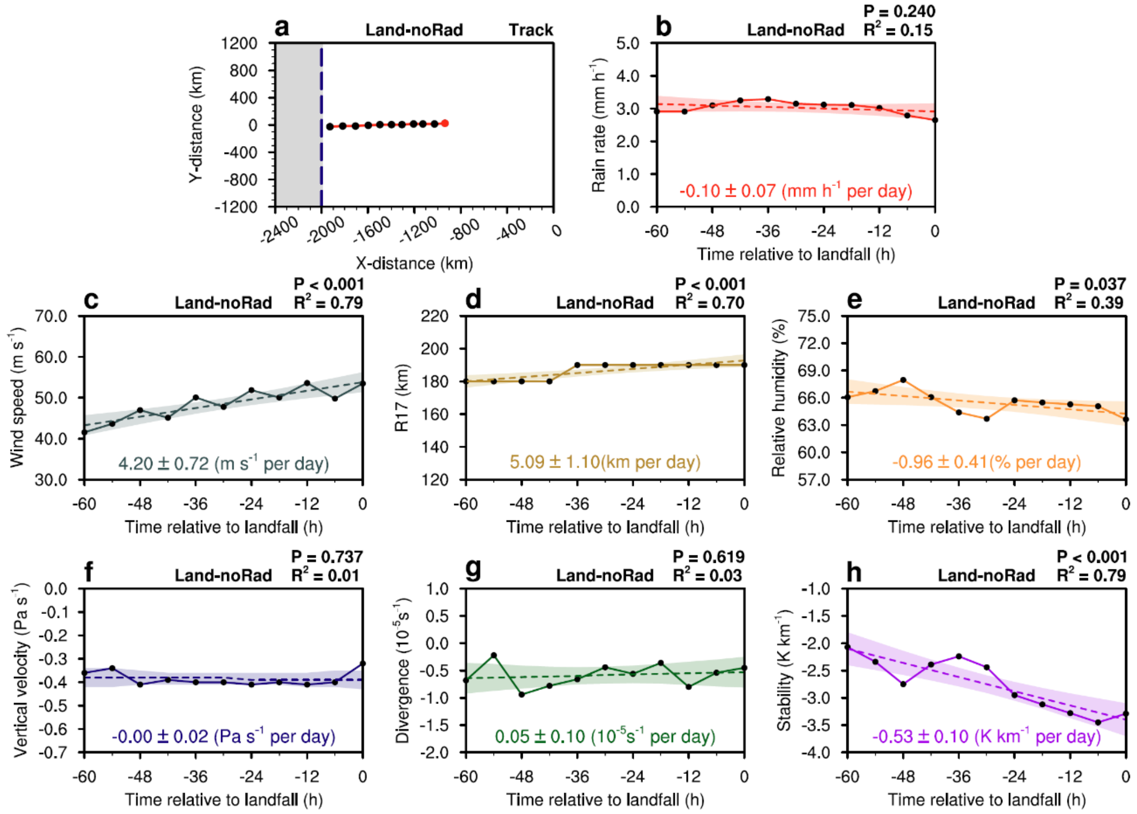

Supplementary Fig. 7 | Track and changes in rain rate, intensity, size and environmental parameters of the simulated Tropical cyclone (TC) in the Experiment 3 (EXP3, Land-noRad). (**a**) 60-hr track, (**b**) rain rate (mm h^{-1}), (**c**) maximum wind speed (m s^{-1}), (**d**) radius of 17 m s^{-1} wind(R17, km), (**e**) relative humidity (%), (**f**) vertical velocity (Pa s^{-1}); (**g**) divergence (10^{-5} s^{-1}), and (**h**) stability (K km^{-1}) of the simulated TC at 6-hourly intervals (solid lines with black dots). Different colors of lines and shading represent different environmental parameters across the entire TC (0 to 500 km radius). In panel (**a**), the starting point is the TC location 60 hours before landfall (red dot) and the blue dashed line indicates the coastline, with land to the west and the sea to the east. Light shading in panels (**b**) to (**h**) represents the 95% confidence interval of the linear trend. Statistical significance of the linear trends was assessed using a Student's t-test. Dashed lines indicate the linear regression of the rain rate, intensity, size and environmental parameters during the time before landfall. Linear regression coefficients with corresponding standard errors are shown at the bottom, and R^2 and p values are provided in the top right corner. Time is presented in hours relative to landfall (00 h), with negative values indicating hours before TC landfall.

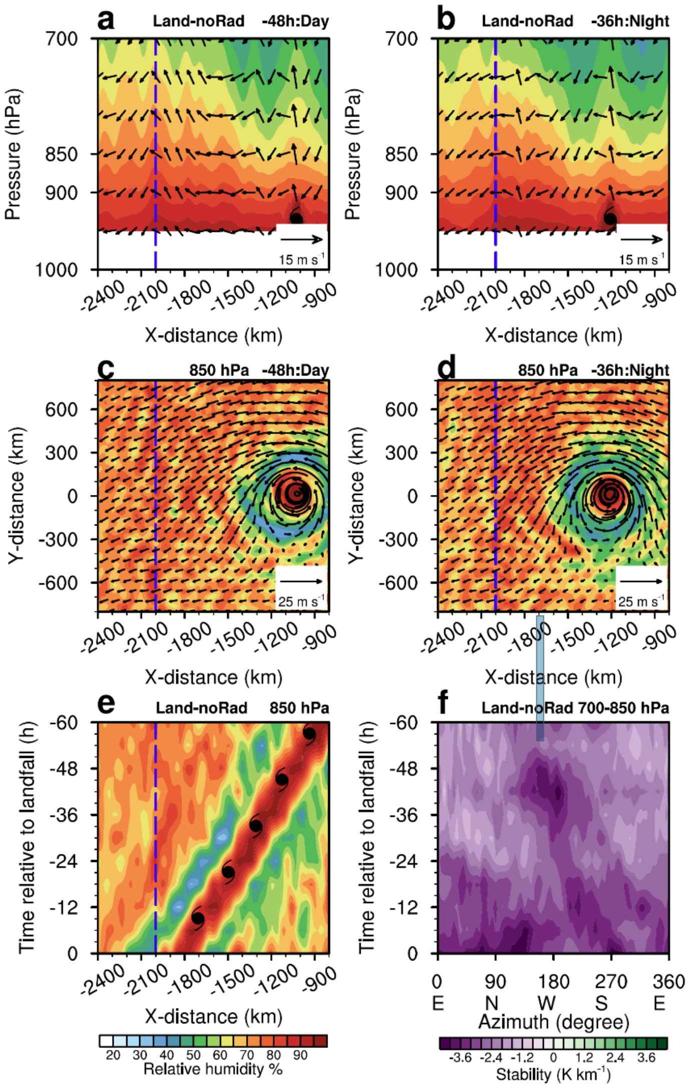

Supplementary Fig. 8 | Spatial and temporal variations of wind vectors (m s^{-1}), relative humidity (shading, %), and stability (K km^{-1}) in the Experiment 3 (EXP3, Land-noRad). (**a**), (**b**) Radius–pressure Hovmöller diagrams of wind and relative humidity at y = 0 km for the TC during the 48 hours of daytime and 36 hours of nighttime before landfall. (**c**), (**d**) horizontal distributions of wind and relative humidity fields at 850 hPa during daytime and nighttime. (**e**), (**f**) azimuthal–time Hovmöller diagrams of respective relative humidity at 850 hPa and stability (the difference in equivalent potential temperature between 850 hPa and 700 hPa). In panels (**a**) to (**d**), the time in the top left corner is in hours relative to landfall (00 h), with negative values indicating hours before landfall. Day and night refer to horizontal and vertical distributions of wind vectors and relative humidity (shading) during the day and night, respectively. In panels (**e**) and (**f**), we averaged the azimuthal–time Hovmöller diagram over the tropical cyclone radius from 0 to 500 km. In panels (**a**) to (**e**), the blue dashed line indicates the coastline, with land to the west and the sea to the east. The radius values on the x- and y-axes are referenced to the model domain center, where negative values represent positions west and south of the center and positive values represent positions east and north of the center. In panels (**a**), (**b**), and (**e**), the typhoon symbol corresponds to the x-axis values of the TC center location. In panel (**f**), the letters E, N, W, and S represent East, North, West, and South in Earth's coordinate system.
